# A hypothermia mimetic molecule (zr17-2) reduces ganglion cell death, gliosis, and electroretinogram distortion in male rats subjected to perinatal asphyxia

**DOI:** 10.3389/fphar.2023.1252184

**Published:** 2023-09-11

**Authors:** Manuel Rey-Funes, Juan Carlos Fernández, Rafael Peláez, Manuel Soliño, Daniela S. Contartese, Nicolás S. Ciranna, Ronan Nakamura, Aníbal Sarotto, Verónica B. Dorfman, José M. Zapico, Ana Ramos, Beatriz de Pascual-Teresa, Juan José López-Costa, Ignacio M. Larrayoz, Alfredo Martínez, César Fabián Loidl

**Affiliations:** ^1^ Laboratorio de Neuropatología Experimental, Instituto de Biología Celular y Neurociencia “Prof. E. De Robertis” (IBCN), Facultad de Medicina, CONICET—Universidad de Buenos Aires, Buenos Aires, Argentina; ^2^ Departamento de Biología Celular, Histología, Embriología y Genética, Instituto de Biología Celular y Neurociencia “Prof. E. De Robertis” (IBCN), Facultad de Medicina, Universidad de Buenos Aires, Buenos Aires, Argentina; ^3^ Biomarkers and Molecular Signaling Group, Center for Biomedical Research of La Rioja (CIBIR), Logroño, Spain; ^4^ Centro de Estudios Biomédicos Básicos, Aplicados y Desarrollo (CEBBAD), Universidad Maimónides, Buenos Aires, Argentina; ^5^ Department of Chemistry and Biochemistry, Facultad de Farmacia, Universidad San Pablo-CEU, CEU Universities, Madrid, Spain; ^6^ Department of Nursing, Biomarkers, Artificial Intelligence, and Signaling (BIAS), University of La Rioja, Logroño, Spain; ^7^ Angiogenesis Group, Oncology Area, Center for Biomedical Research of La Rioja (CIBIR), Logroño, Spain

**Keywords:** hypothermia, perinatal asphyxia, hypothermia mimetics, apoptosis, electroretinogram, cold-shock proteins, gliosis

## Abstract

**Introduction:** Perinatal asphyxia (PA) represents a major problem in perinatology and may cause visual losses, including blindness. We, and others, have shown that hypothermia prevents retinal symptoms associated to PA. In the present work, we evaluate whether a hypothermia mimetic small molecule, zr17-2, has similar effects in the context of PA.

**Methods:** Four experimental groups were studied in male rats: Naturally born rats as controls (CTL), naturally born rats injected s.c. with 50 µL of 330 nmols/L zr17-2 (ZR), animals that were exposed to PA for 20 min at 37°C (PA), and rats that were exposed to PA and injected with zr17-2 (PA-ZR). Forty-five days after treatment, animals were subjected to electroretinography. In addition, morphological techniques (TUNEL, H&E, multiple immunofluorescence) were applied to the retinas.

**Results:** A reduction in the amplitude of the a- and b-wave and oscillatory potentials (OP) of the electroretinogram (ERG) was detected in PA animals. Treatment with zr17-2 resulted in a significant amelioration of these parameters (*p* < 0.01). In PA animals, a large number of apoptotic cells was found in the GCL. This number was significantly reduced by treatment with the small molecule (*p* < 0.0001). In a similar way, the thickness of the inner retina and the intensity of GFAP immunoreactivity (gliosis) increased in PA retinas (*p* < 0.0001). These parameters were corrected by the administration of zr17-2 (*p* < 0.0001). Furthermore, injection of the small molecule in the absence of PA did not modify the ERG nor the morphological parameters studied, suggesting a lack of toxicity.

**Discussion:** In conclusion, our results indicate that a single s.c. injection of zr17-2 in asphyctic neonates may provide a novel and efficacious method to prevent the visual sequelae of PA.

## 1 Introduction

Despite the progress in obstetrics and perinatology, perinatal asphyxia (PA) is still a significant health challenge (McGuire, 2007; Cunningham et al., 2018). PA results from a lack of oxygen secondary to interruption of placental blood flow while the fetus is still in the birth canal and thus unable to transition to pulmonary respiration. This interruption of oxygenation causes fetal hypoxia, hypercarbia, and acidosis. If the hypoxic insult becomes severe and/or prolonged enough, the fetus may suffer hypoxic ischemic injury, promoting neural cell death (Rainaldi and Perlman, 2016). Most cases of PA are related to intrapartum events but underlying chronic maternal and fetal conditions must be considered, as well, allowing the identification and prevention of high-risk pregnancies (Herrera and Silver, 2016).

PA may generate diverse central nervous system (CNS) sequelae (Volpe, 1987). The most common damage associated to PA are epilepsy, attention deficit hyperactivity disorder, spasticity, intellectual disability, and auditory or visual alterations (Crofts et al., 1998; Herrera et al., 2018). Therefore, an early assessment of the severity of the damage is peremptory to perform timely interventions (Walas et al., 2020).

Different animal models of PA have been developed. Perhaps, the model that best recapitulates the human condition was described by our group about 30 years ago, and consists on the incubation of the end-term fetuses, still inside the uterus, in a water bath for a predetermined time (Loidl et al., 1994). In this model, the animals develop lesions in the brain, the spinal cord, and the eyes (Loidl et al., 1994; Dorfman et al., 2009). In the eye, PA produces different neurological injuries, including retinopathies (Rey-Funes et al., 2010). The hypoxic/ischemic retinas are characterized by modifications of the electroretinogram (ERG) (Rey-Funes et al., 2021), pathological angiogenesis, gliosis, and ganglion cell death, resulting in a significant thickening of the inner retina (Rey-Funes et al., 2013).

The current gold standard to treat PA is therapeutic hypothermia (Thoresen, 2015; Rivero-Arias et al., 2019). The mechanism of action of therapeutic hypothermia was initially postulated as a general decrease in the metabolism, including reduced enzymatic activity. Nowadays, additional molecular mechanisms have been proposed (Al-Fageeh and Smales, 2006). A small family of proteins has been identified whose expression increases when the body temperature drops and, therefore, are known as cold-shock proteins. Two main proteins of this family are found in mammals; cold-inducible RNA binding protein (CIRP) and RNA binding motif protein 3 (RBM3) (Chip et al., 2011; Tong et al., 2013). CIRP and RBM3 work as chaperones for specific RNAs and regulate their translation and function (Lleonart, 2010; Wellmann et al., 2010; Liu et al., 2013). CIRP and RBM3 are found both in the brain (Schmitt et al., 2007; Tong et al., 2013) and retina (Larrayoz et al., 2016) of mammals.

Usually, newborns requiring therapeutic hypothermia treatment are exposed to the cold for several days (Azzopardi et al., 2008). This prolonged treatment could generate unexpected complications, including extreme hypothermia, hypoglycemia, bradycardia, skin necrosis, sepsis, systemic hypotension, or pulmonary hypertension (Saw et al., 2019). Therefore, alternative methods may reduce these undesired complications. To provide such an alternative, we have developed a group of small molecules that bind to CIRP and increase its protein half-life, probably through inhibition of a yet unknown protease (Coderch et al., 2017). One of these molecules, zr17-2, has been used successfully to prevent retinal injury secondary to optic nerve traumatism (Contartese et al., 2023).

The aim of this study was to demonstrate the beneficial effects of a single subcutaneous (s.c.) injection of zr17-2 in newborns affected by PA, as they relate to different hallmarks of retinal function and morphology.

## 2 Materials and methods

### 2.1 Perinatal asphyxia model

**FIGURE 1:**
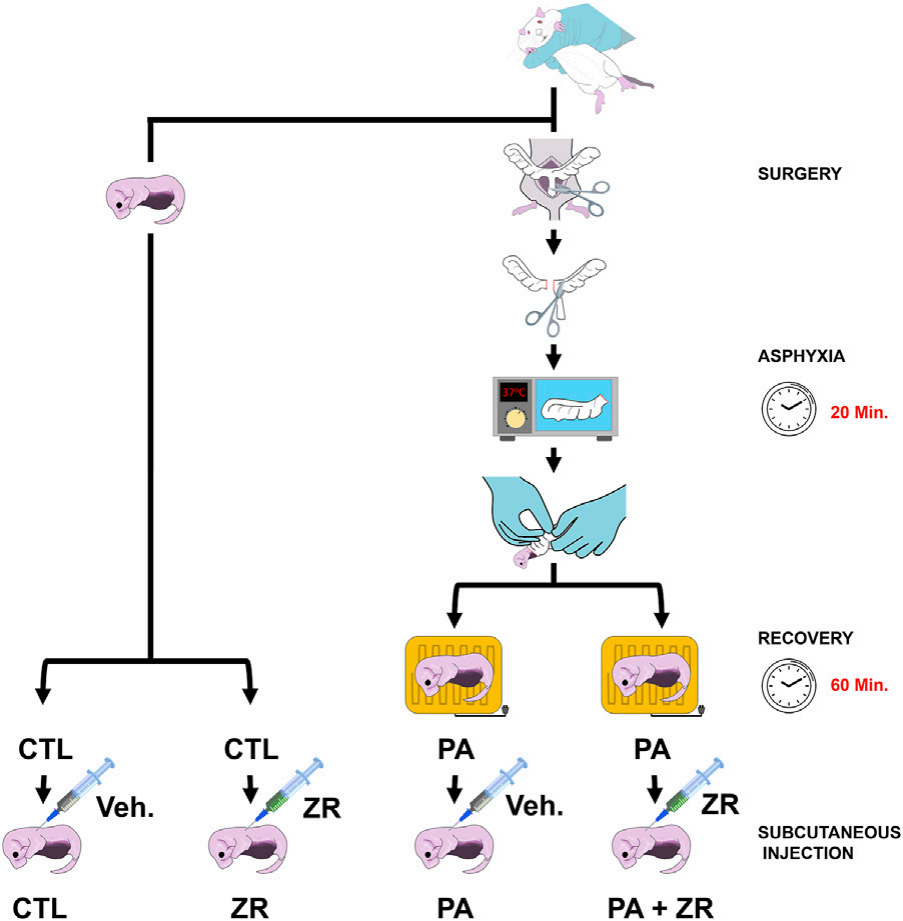
Schematic drawing of the experimental procedure. Normally delivered pups were assigned to the control groups. Perinatal asphyxia (PA) was generated by introducing the uterine horns in a water bath at 37°C for 20 min. Half of the animals were injected with zr17-2 and the other half with vehicle to generate the four experimental groups.

Sprague-Dawley albino rats were provided by the animal facility of our institution. All procedures were approved by the Ethical Committee of CICUAL (Comité Institucional para el Uso y Cuidado de Animales de Laboratorio, Resolution Nº 3021/2019), Facultad de Medicina, Universidad de Buenos Aires, Argentina. Appropriate proceedings were performed to minimize the number of animals used and their suffering, pain, and discomfort. PA was induced as previously described (Loidl et al., 1998; Fernandez et al., 2020). Briefly, term pregnant rats were sacrificed after their first pup was born and hysterectomized. Normally delivered newborns were used as controls (CTL group). The uterus was immersed in a water bath at 37°C for 20 min to generate the ischemic insult (PA group). Pups were removed from the uterine horns and placed under a heating lamp for 1 h. To avoid the influence of hormonal variations due to the female estrous cycle (Chaychi et al., 2015), only male pups were included in this study. One hour later, animals were randomly divided in two groups, and received a 50 μL s.c. injection of either 330 nmols/L zr17-2 or phosphate saline buffer (PBS), as vehicle (Figure 1). This dose was chosen based on previous studies that showed drug efficacy (Coderch et al., 2017; Contartese et al., 2023).

The small molecule zr17-2 was initially synthesized in house in the search of new CK2 inhibitors. This compound is a purine derivative which mimics the adenine present in ATP, and therefore may act as an ATP competitive kinase inhibitor. Although docking studies predicted high affinity for CK2, a radiometric enzymatic assay revealed that the compound was inactive against this enzyme (Maslyk et al., 2010). Some years later, compound zr17-2 was identified as a hypothermia mimetic using a High Throughput Virtual Screening (HTVS) on diversity set IV of the NCI and fifteen molecules of our database (Coderch et al., 2017).

### 2.2 Tissue collection

Animals were sacrificed depending on the requirements of each technique. Lethal anesthesia was implemented by i.p. injection of 300 mg/Kg ketamine (Imalgene, Merial Laboratorios, Barcelona, Spain) plus 30 mg/Kg xylazine (Xilagesic, Proyma Ganadera, Ciudad Real, Spain). The eyes were enucleated, fixed in 4% paraformaldehyde in 0.1 M pH 7.4 phosphate buffer at 4 °C for 48h, dehydrated, and paraffin embedded.

### 2.3 TUNEL assay

Five-day-old rats (*n* = 5 animals per group) provided paraffin-embedded eyes for the TUNEL assay, as described (Contartese et al., 2023). Serial sections (5 μm thick) were mounted onto coated slides. The *In Situ* Cell Death Detection POD Kit (Roche, Basel, Switzerland) was used to detect apoptotic cells, following manufacturer´s instructions. Incubation with 10 IU/mL DNaseII (Sigma Chemical Co.) in 50 mM Tris–HCL, pH 7.5, 10 mM Mg_2_Cl, and 1 mg/mL BSA, for 10 min at room temperature was used as a positive control. Omission of the deoxynucleotidyl transferase enzyme was used as a negative control.

### 2.4 H&E and immunofluorescence

After animals were subjected to electroretinography (see below), they were deeply anaesthetized, their eyes were enucleated, fixed, and paraffin-embedded, as above (*n* = 5 animals per group). Tissue sections (5 µm-thick) were dewaxed and rehydrated through graded ethanol. Some sections were stained with hematoxylin and eosin to establish morphological parameters. Others were subjected to immunofluorescence for markers of gliosis. For that purpose, slides were incubated with a blocking solution containing 10% normal donkey serum in PBS (pH 7.4) for 30 min. Then a a rabbit polyclonal anti-GFAP antibody (dilution 1:500; Dako, Glostrup, Denmark) was added to the slides and incubated at 4°C overnight. Next day, after three washes in PBS, slides were incubated with Alexa fluor 555-donkey-anti-rabbit IgG (dilution 1:200, Thermo Fisher Scientific, MA, United States) for 1 h at room temperature. After another set of three washes in PBS, nuclei were counterstained with 40-6-diamidino-2-phenylindole (DAPI) (1:1000; Sigma). The thickness of the inner retina, which includes the internal limiting membrane, the retinal optic nerve fiber layer, and the ganglion cell layer (GCL), as reported (Rey-Funes et al., 2013), was measured in cross-sections of the posterior segments of the eye. A BX40 Olympus microscope (Tokyo, Japan) with a 390CU 3.2 Megapixel CCD camera (Micrometrics, Spain) was used to take histological images. A Nikon C1 Plus (Tokyo, Japan) confocal microscope was used to capture immunofluorescent images.

### 2.5 Image analysis

Image analysis was performed as reported (Contartese et al., 2023). Briefly, several fields (*n* = 10) were chosen in the central region of each retina under the ×40 microscope objective, and the number of cells that exceeded the established threshold (defined as the optic density 3-fold higher than the mean background density) were counted. Micrometrics SE P4 (Standard Edition Premium 4, Micrometrics, Spain) software was used for this purpose. In addition, digital manipulation of brightness and contrast of the images was performed with Adobe Photoshop software (Adobe Photoshop CS5, Adobe Systems Inc., Ottawa, ON, Canada).

### 2.6 Electroretinograms

Forty-five-day-old rats (*n* = 9 per experimental group) were studied by electroretinography, as described (Rey-Funes et al., 2017). Briefly, animals were placed at a distance of 25 cm from the light source. Reference electrodes were placed in the ear and the tail, while a gold electrode was touching the cornea. Scotopic electroretinograms (ERG) from both eyes were recorded in response to recurrent flashes of light (1 ms, 1 Hz). The registered response was amplified (9 cd s/m^2^ without filter), filtered (1.5-Hz low-pass filter, 500 Hz high-pass filter, notch activated), and averaged (Akonic BIO-PC, Buenos Aires, Argentina). To identify oscillatory potentials (OP), filters of high (300 Hz) and low (100 Hz) frequency were included. Values from both eyes were averaged, and data are presented as mean ± standard error of the mean (SEM).

### 2.7 Statistical analysis

Normally distributed data were evaluated by one way ANOVA followed by the Dunnet’s (Bonferroni) or Holm-Sidak post-hoc test while data not following a normal distribution were analyzed with the Kruskal–Wallis test followed by Dunn´s test. All data were analyzed with GraphPad Prism 8.0 software and were considered statistically significant when *p* < 0.05.

## 3 Results

### 3.1 Small molecule zr17-2 restores electroretinogram patterns

Animals were randomly divided into the four experimental groups depending on whether they were subjected to PA and/or zr17-2 injection (Figure 1). All animals were examined daily after treatment and none of them experienced any apparent adverse reaction to the small molecule.

**FIGURE 2:**
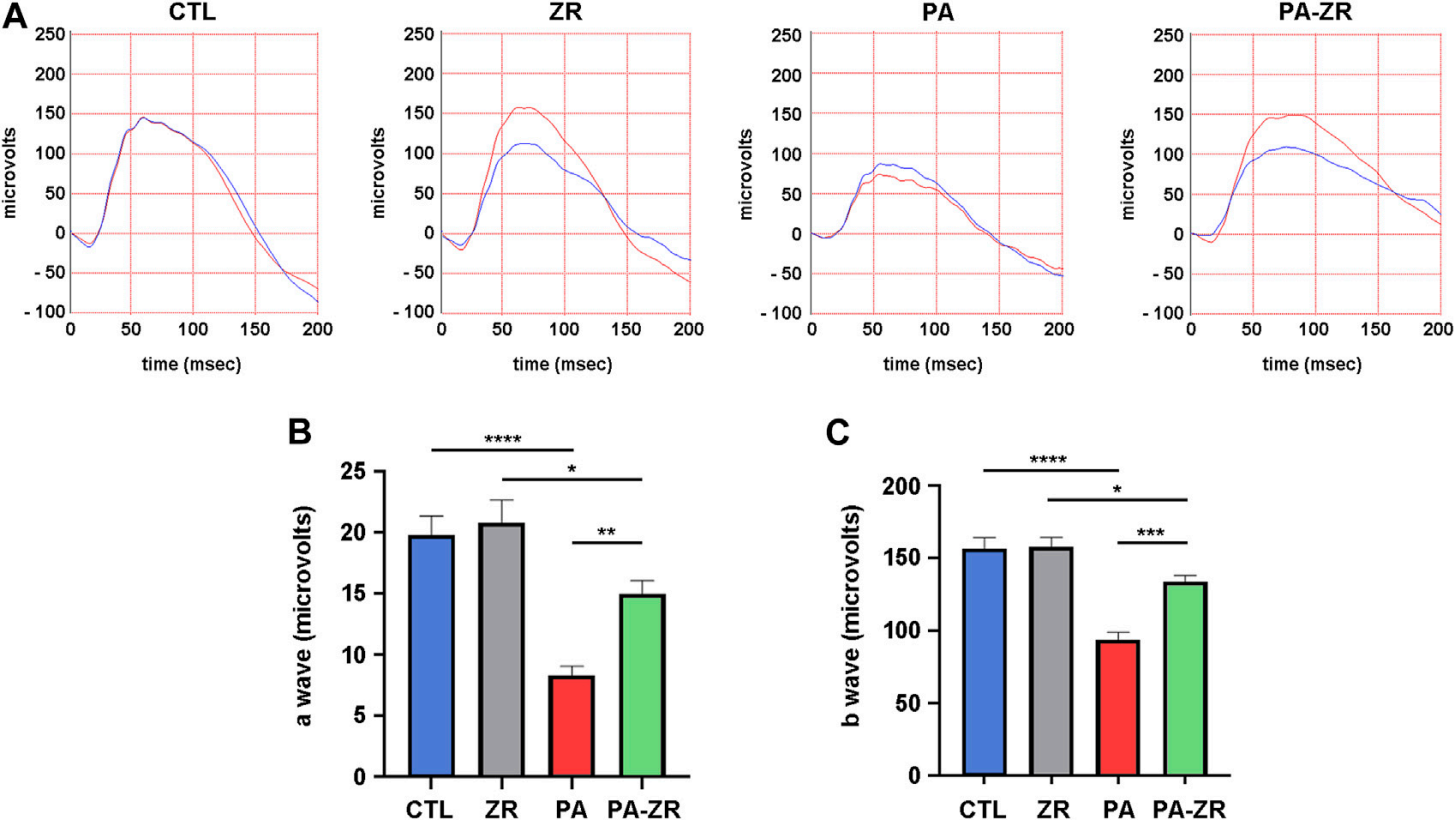
Small molecule zr17-2 prevents changes in the electroretinogram induced by perinatal asphyxia. **(A)** Representative electroretinograms of 45 day-old animals (*n* = 9 per group) subjected to PA with and without small molecule injection. The red line corresponds to the right eye whereas the blue line is the recording of the left eye. **(B)** Amplitude of the a-wave in the four experimental groups. **(C)** Amplitude of the b-wave in the four experimental groups. In both cases, perinatal asphyxia (PA) induced a significant decrease in the a- and b-wave compared to control (CTL), whereas the small molecule partially prevented it. Each bar represents the mean ± SEM of 9 animals. Two way ANOVA. Asterisks indicate significant differences. *:*p* < 0.05; **:*p* < 0.01; ***:*p* < 0.001; ****:*p* < 0.0001.

**FIGURE 3:**
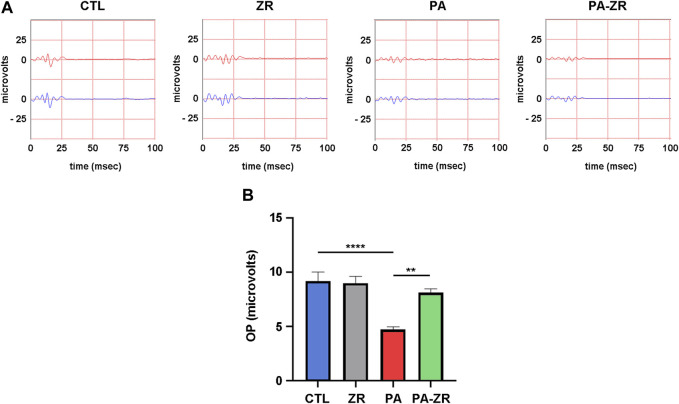
Small molecule zr17-2 prevents changes in the oscillatory potentials induced by perinatal asphyxia. **(A)** Representative oscillatory potentials (OP) of the electroretinograms of 45 day-old animals (*n* = 9 per group) subjected to PA with and without small molecule injection. The red line corresponds to the right eye whereas the blue line is the recording of the left eye. **(B)** Sum of amplitudes of the OP in the four experimental groups. Perinatal asphyxia (PA) induced a significant decrease in the OPs compared to control (CTL), whereas the small molecule prevented it. Each bar represents the mean ± SEM of 9 animals. Two way ANOVA. Asterisks indicate significant differences. **:*p* < 0.01; ****:*p* < 0.0001.

Forty-five days after treatment, scotopic ERG was performed in all rats. As described in previous studies (Rey-Funes et al., 2021), animals subjected to the PA procedure showed a significant decrease in the amplitude of the a-waves (Figures 2A, B), the b-waves (Figures 2A, C), and the OPs (Figure 3). Administration of zr17-2 to normally delivered rats had no implications in the ERG profile (Figures 2, 3). Interestingly, though, treatment with zr17-2 produced a significant recovery of the a-wave (*p* < 0.01), the b-wave (*p* < 0.001) (Figures 2B, C), and oscillatory potentials (*p* < 0.01) (Figure 3B) in animals affected by PA.

### 3.2 Small molecule zr17-2 reduces PA-mediated apoptosis in the retina

**FIGURE 4:**
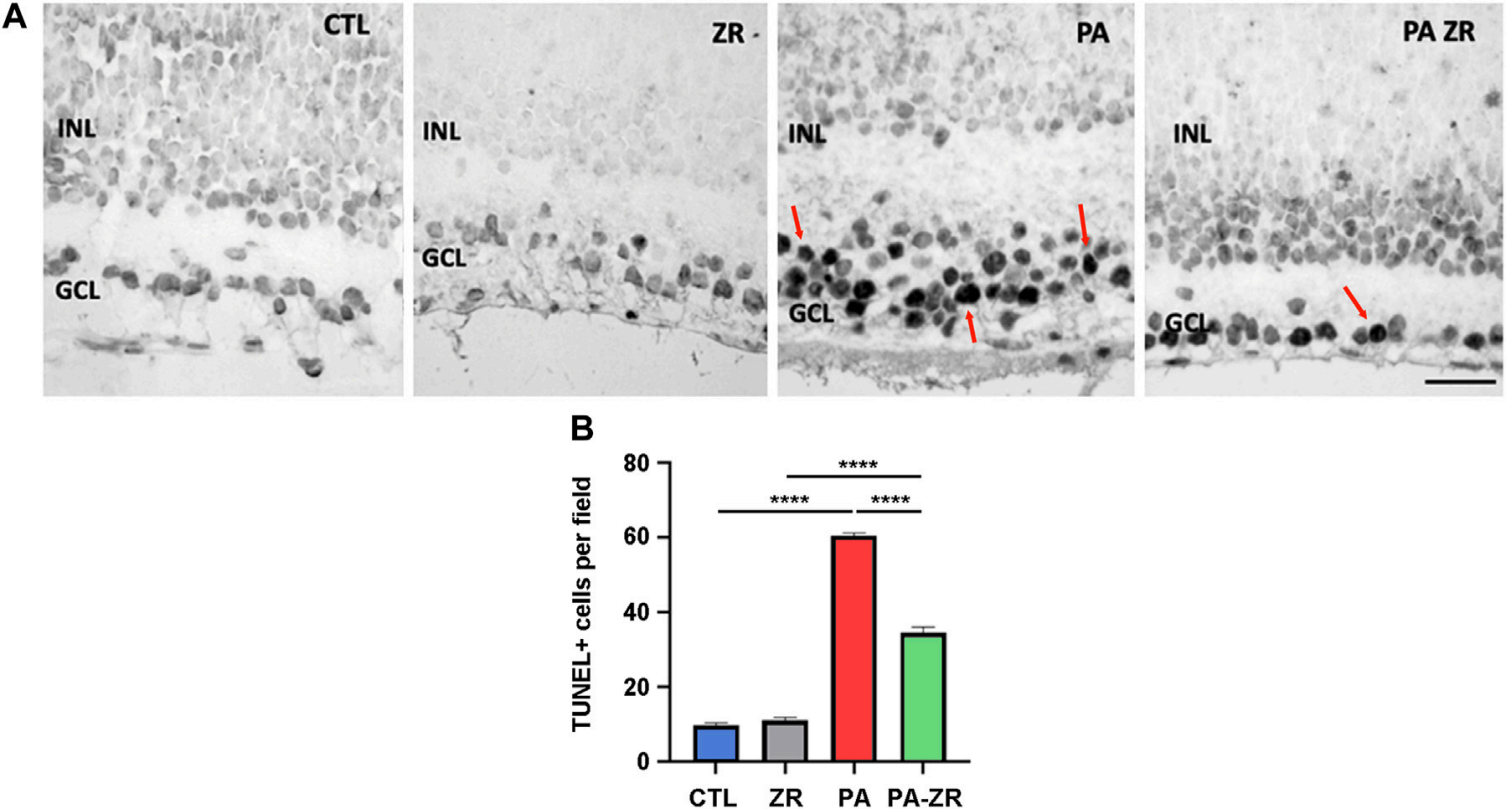
Apoptosis induced by perinatal asphyxia is prevented by treatment with the small molecule. Representative images of apoptotic cells (red arrows) localized in the ganglion cell layer as labeled by the TUNEL assay in the four experimental groups **(A)**. Scale bar: 40 µm. Graphical representation of TUNEL-positive cell number **(B)**. The PA group showed a significant increment in the number of TUNEL positive cells compared to the CTL group. The small molecule (PA-ZR) significantly prevented apoptosis induction. Two way ANOVA. Each bar represents the mean ± SEM of 5 animals. Asterisks indicate significant differences. ****:*p* < 0.0001.

The number of TUNEL-positive cells in the GCL of PA animals was much higher (about 6-fold) than in the CTL group (*p* < 0.0001) (Figure 4). This number got drastically reduced by injection of zr17-2 (*p* < 0.0001). Unfortunately, the number of apoptotic cells was still far from the basal levels (*p* < 0.0001). We should notice that administration of zr17-2 did not modify the number of TUNEL-positive cells in the ZR group, suggesting a lack of toxicity for this small molecule (Figure 4).

### 3.3 Small molecule zr17-2 prevents PA-induced inner retina thickening

**FIGURE 5:**
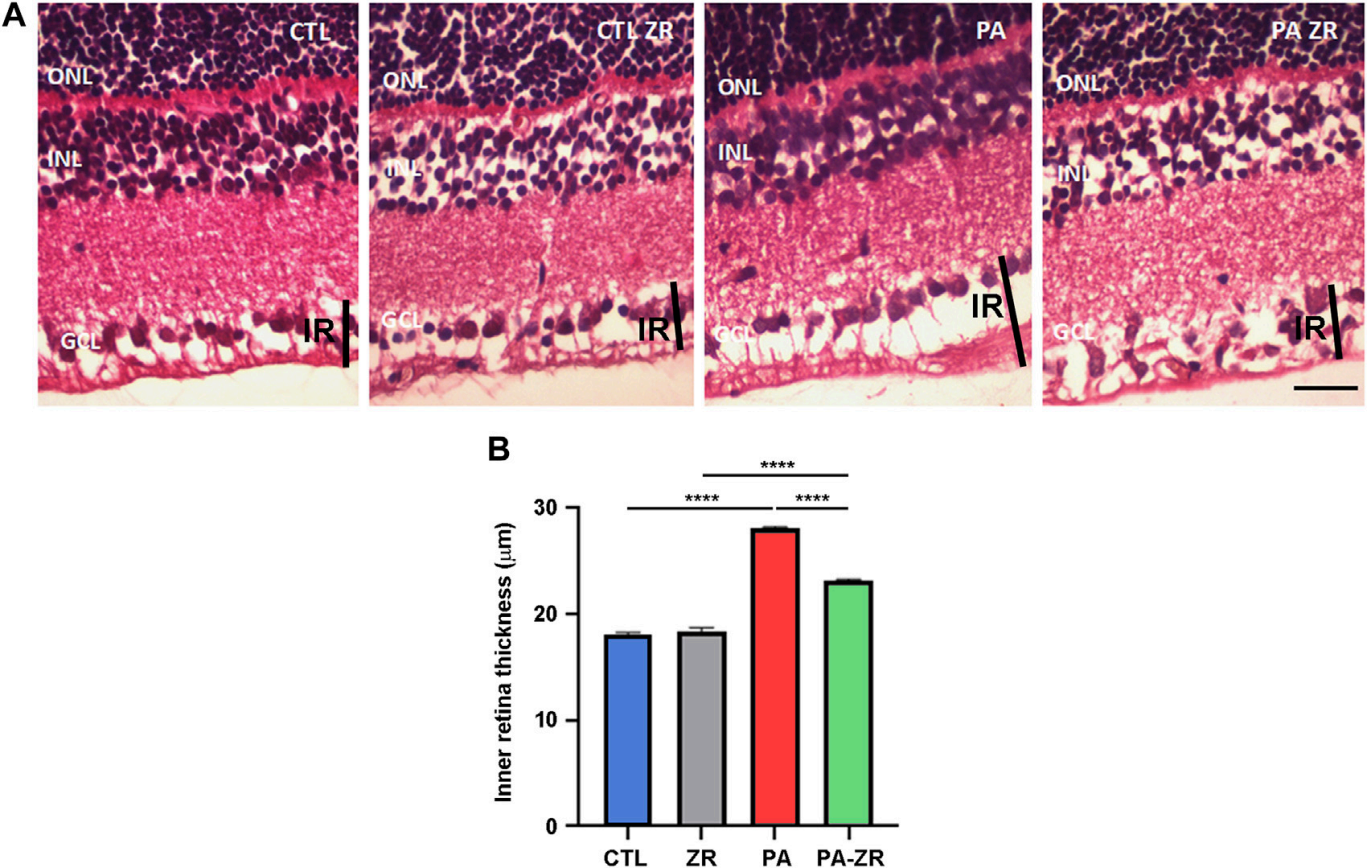
Inner retina thickening is prevented by the small molecule. Representative histological images of the retina of animals of the 4 experimental groups, taken 45 days after birth, and stained with hematoxylin-eosin **(A)**. Three layers of the retina are labeled in the pictures for reference: outer nuclear layer (ONL), inner nuclear layer (INL), and ganglion cell layer (GCL). A black vertical bar demarcates the inner retina (IR). Scale bar = 50 μm. Quantification of the IR thickness is shown as a histogram **(B)**. Bars represent the mean ± SEM of all samples (*n* = 5 animals per group, 4 measurements per animal). Asterisks represent statistically significant differences. ****p < 0.0001. Statistical test: Two way ANOVA followed by Holm-Sidak post-hoc test.

According to previous reports (Rey-Funes et al., 2013), the inner retina was defined as the area encompassing the internal limiting membrane, the retinal optic nerve fiber layer, and the GCL. Our morphological data show that PA translates into an increase on thickness of the inner retina (*p* < 0.0001) (Figure 5). Injection of zr17-2 significantly (*p* < 0.0001) reduced this parameter, although this value was still higher than the controls (*p* < 0.0001) (Figure 5). As above, administration of zr17-2 in normally delivered rats produced no changes in the thickness of the inner retina (Figure 5).

### 3.4 Small molecule zr17-2 reduces PA-induced gliosis

**FIGURE 6:**
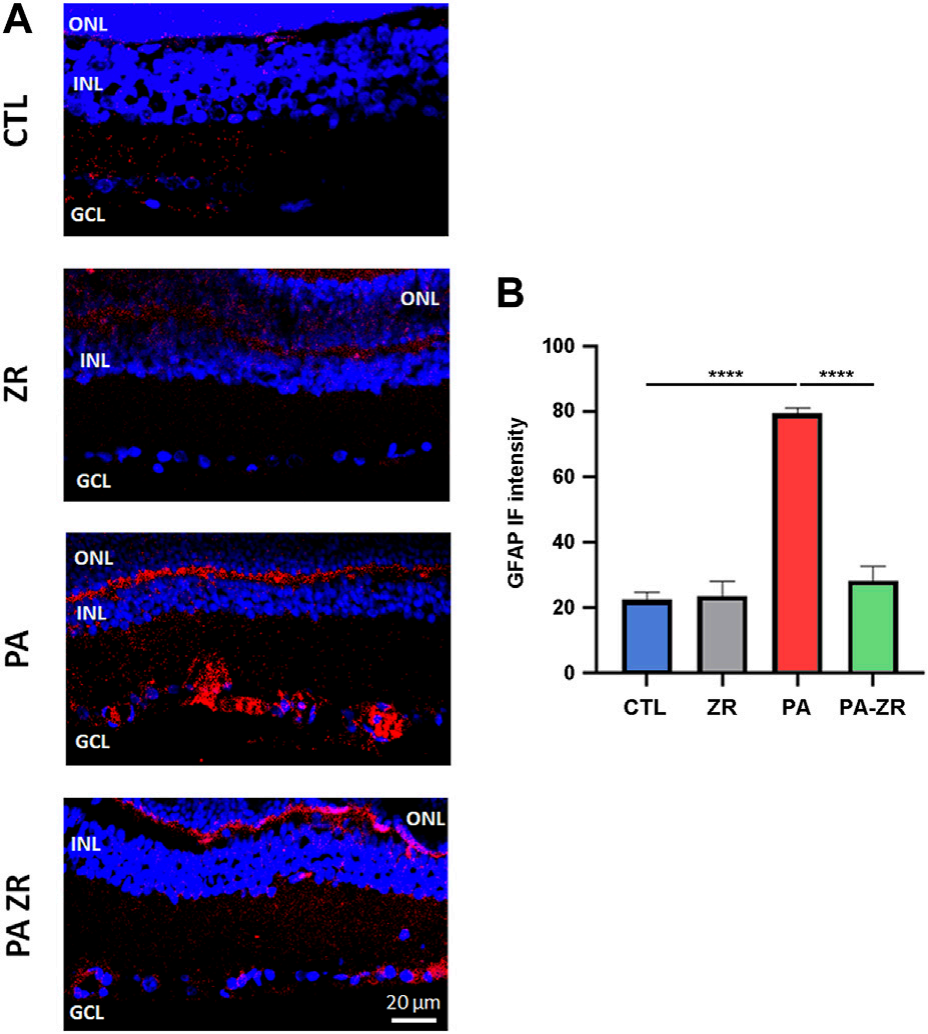
The small molecule prevents gliosis. Representative confocal microscopy images, labeled with an antibody against GFAP (red), of the retina of animals of the 4 experimental groups taken 45 days after birth. Nuclei were counterstained with DAPI (blue) **(A)**. Three layers of the retina are labeled in the pictures for reference: outer nuclear layer (ONL), inner nuclear layer (INL), and ganglion cell layer (GCL). Size bar = 20 μm. Graphical representation of the intensity of GFAP staining in the inner retina **(B)**. Two way ANOVA. Each bar represents the mean ± SEM of 5 animals. Asterisks indicate significant differences. ****:*p* < 0.0001.

Previous studies found that PA results in a pathological increase of angiogenesis and gliosis in the retina (Rey-Funes et al., 2013). To investigate this issue, we stained retina sections with an antibody against GFAP to label the processes of Müller cells (Figure 6A). As expected, a significant increase in the immunoreactivity for GFAP in the outer plexiform layer, the GCL, and the inner limiting membrane (*p* < 0.0001) was elicited by PA (Figure 6B). Treatment of the pups with zr17-2 significantly reduced gliosis (*p* < 0.0001) to the point that these values are statistically undistinguishable from the control animals (Figure 6B).

## 4 Discussion

In this study, we have shown that a single dose of zr17-2 injected in newborns exposed to PA was able to significantly reduce all the physiological and morphological tell tales of ischemia-induced retinopathy. These included perturbations of the ERG, number of apoptotic cells in the GCL, thickness of the inner retina, and gliosis. Furthermore, injection of zr17-2 on normally delivered pups had no effect on any of the measured parameters, suggesting a very desirable lack of toxicity by this molecule.

This study presents some limitations. First, only male rats were used to reduce data set variability. Nevertheless, it will be important to investigate the influence of sex on the studied parameters since clear sex differences in ERG recordings have been described in Sprague-Dawley rats (Chaychi et al., 2015). In addition, different concentrations of the drug, different routes of administration, and different times for the injection of the small molecules after birth should be experimentally tested to better characterize the effects of the hypothermia mimetic small molecules on PA. Furthermore, other techniques, including OCT to measure retinal thickness, could provide additional information on the protective effects of the small molecules.

In this study, we have employed an animal model of PA in rats based on our previous long experience (Loidl et al., 1994; Dorfman et al., 2009). Some animal models that have been proven crucial for perinatal brain and ocular research include non-human primate fetuses and neonates, as well as pregnant sheep, lambs, puppies, piglets, and immature rodents (Mota-Rojas et al., 2022). In the case of rats and mice, the developmental stage of rodent newborns can be compared with that of premature human infants, so this should be taken into consideration when trying to translate our data into the clinic. Although no model is perfectly ideal in terms of capturing the diversity and complexity of human brain/eye pathology, the investigator must evaluate the strengths and limitations of each model in the context of the research questions, and future studies in alternative animal models, and especially the use of primates, should reinforce our current data.

Previous electroretinographic studies have shown that PA results in a reduction of the amplitudes of the a-wave, the b-wave, and the OPs (Fernandez et al., 2020; Rey-Funes et al., 2021). Our current data confirm these previous observations. The a-wave measures the electric activity of the photoreceptors, whereas the b-wave corresponds to the ganglion cells’ function, and the OPs to the activity of the neurons whose nuclei are located in the inner nuclear layer (INL), namely, the bipolar, horizontal, and amacrine neurons (Jung et al., 2015; Matei et al., 2020; Zhai et al., 2020). Therefore, a drastic reduction in the three components of the ERG clearly indicates a compromised function of the whole visual pathway, which correlates with a drastically reduced visual acuity (Rey-Funes et al., 2017). ERG is not commonly performed in asphyctic neonates in the clinic but electroencephalograms (EEG) are usually recorded. Interestingly, PA induces a reduction in the amplitude of the EEG bursts revealing a distortion in the electrical activity of the brain (Liu et al., 2016), in a similar fashion to our observations in the retina. Quantification of this EEG amplitude reduction is a good predictor of long-term neurological outcome for affected neonates (Han et al., 2019). Our results have shown that a single injection of zr17-2 within the first hours after birth was able to significantly prevent part of the reduction in amplitude for the three components of the ERG. This prevention was complete for the OPs but partial for the a- and b-waves. Future studies will investigate whether a higher concentration of the molecule or a series of injections would improve the effects of zr17-2 on the ERG. In addition, taking into consideration the similarities between the physiology of the retina and the brain, it should be interesting to investigate whether zr17-2 has a positive effect on preventing the CNS complications associated to PA. A very encouraging observation was the lack of effects of the small molecule on normally delivered neonates (group ZR), indicating a lack of toxicity on the visual pathway.

We performed the ERG procedure 45 days after birth since by this time the rats have reached sexual maturity and can be compared to human young adults (Sengupta, 2013). Therefore, by this stage, the remaining visual damage may not be recoverable and studying the ERG at longer times may not provide any further difference. Interestingly, changes in b-wave amplitude correlated with a large number of apoptotic cells in the GCL whereas no significant numbers of apoptotic cells were detected in the ONL or the INL despite the changes in the a-wave and the OPs. In fact, all the studies performed on PA have made the same observation (Fernandez et al., 2020; Rey-Funes et al., 2021). This is probably due to the fact that the electric currents traveling through the neuronal path of the retina can be influenced by a number of pathophysiological stimuli which do not need, necessarily, to result in neuronal death.

The small molecule was injected subcutaneously 1 hour after retrieving neonates from the hypoxic uterus. This route of administration was chosen because it provides an easy way of delivering the molecules with little discomfort for the neonates. In adult animals, it has been shown that zr17-2 does not cross the blood-brain barrier nor the blood-retinal barrier (Coderch et al., 2017). We took advantage of the fact that these barriers are not fully functional in newborn animals (Chow and Gu, 2017) and used the subcutaneous route of administration, which is easier and less traumatic for the newborns than the intravitreal one. Regarding the time between birth and injection of the small molecule, we chose 1 hour because this is approximately the time it takes for the newborn pups to recover from the ischemic shock, allowing the researchers to select the pups that fulfill the inclusion criteria (occipitocaudal length >41 mm, weight >5 g, healthy respiratory frequency, good motility, vocalization, and healthy skin color) and discard those who do not. Furthermore, 1 hour is a realistic time in clinical practice to allow practitioners to perform required PA (Apgar) tests on the neonates, confirm the diagnosis, and obtain a signed informed consent from the parents. Nevertheless, future studies should investigate the maximum period at which the small molecules are still functional to prevent PA-related symptoms.

In parallel to the ERG data, a 6-fold increase in the number of apoptotic cells in the GCL was produced by PA, in agreement with previous reports (Fernandez et al., 2020; Rey-Funes et al., 2021). Histological considerations indicate that a great majority of these cells correspond with ganglion cells. The induction of apoptosis in ganglion cells is a consequence of the hypoxia/ischemia-reperfusion process that occurs in PA (Kaur et al., 2008). This process generates a number of free radicals, including reactive oxygen and nitrogen species. Of these, nitric oxide (NO) is regarded as the main mediator of PA pathophysiology (Rey-Funes et al., 2011). High levels of NO react with superoxide to form peroxynitrite, a powerful oxidant. Peroxynitrite causes oxidation of sulfhydryl groups, lipid peroxidation, RNA and DNA damage, and nitration of tyrosine residues in proteins, all of which induce neuronal apoptosis (Rodrigo et al., 2005). In agreement with the ERG data, small molecule zr17-2 was able to partially prevent retinal cell apoptosis. Since zr17-2 was selected for its ability to increase CIRP levels (Coderch et al., 2017), we can hypothesize that the mechanism of action includes the protection by CIRP of mRNAs related with neuronal survival, which eventually would tilt the scales back from apoptosis to survival (Saito et al., 2010; Chip et al., 2011). Furthermore, a recent study has shown that zr17-2 is also able to reduce oxidative stress damage caused by aluminum maltolate, a potential environmental toxicant that induces ER stress and neuronal ferroptosis (Contartese et al., 2023). As with the ERG data, injection of the small molecule in non-asphyctic neonates had no effect on the number of apoptotic retinal cells.

The thickening of the inner retina has been described as a common manifestation of hypoxia/ischemia-reperfusion in the eye and seems to be due to pathological increases in angiogenesis and gliosis (Rey-Funes et al., 2013; Luo et al., 2018). Müller cells generate additional cell processes in response to hypoxia/ischemia-reoxygenation, that can be followed by their immunoreactivity to GFAP, resulting in generalized gliosis (Pekny et al., 2014). On the other hand, a pathological proliferation of blood vessels occurs through a hypoxia-mediated overexpression of proangiogenic factors and a downregulation of anti-angiogenic molecules within the eye (Friedlander, 2007). In this study, we have confirmed that PA was responsible for an increase in inner retina thickness, concomitant with a pathological proliferation of Müller cell inner processes. All these characteristics were significantly corrected by administration of zr17-2, indicating the great potential of this small molecule. We should note that this increase in inner retina thickness caused by gliosis was larger than the thinning of this layer expected from the apoptosis of ganglion cells demonstrated by TUNEL. Obviously, there is a time factor involved since apoptosis was seen at day 5 post asphyxia whereas the thickening of the inner retina was measured at 45 days.

In conclusion, small molecule hypothermia mimetics offer a new therapeutic avenue to prevent the symptoms of ocular conditions related with hypoxia/ischemia-reperfusion. In the case of PA, therapeutic hypothermia is currently the gold standard (Rivero-Arias et al., 2019) but the right application of the technique requires specialized equipment with high cost (Akula et al., 2015; Dingley et al., 2015). Unfortunately, these costs could be prohibitive for low- and middle-income countries which, by the way, are the places where 96% of all PA cases occur (Mukhtar-Yola et al., 2018). In this context, a single injection of a hypothermia mimetic drug could offer a welcome and more cost-efficient alternative. Obviously, before these drugs can be used in the clinic, regulatory preclinical and clinical development must be completed.

## Data Availability

The raw data supporting the conclusion of this article will be made available by the authors, without undue reservation.
